# Appendix to Braithwaite’s Retrospect

**Published:** 1866-05

**Authors:** 


APPENDIX TO BRAITHWAITE'S RETROSPECT.—The publisher of the American edition of *Braithwaite's Retrospect of Practical Medicine and Surgery* has been long and urgently solicited by many of the leading members of the medical profession in this country to add an Appendix, containing a summary of the important medical features of American journalism, and thus to render the work more acceptable to its readers generally.

Our medical journals contain articles of great value, which do not receive the attention they deserve, thus depriving the profession of the valuable information, and the magazine and the author the reputation that should justly accrue to them. The publisher anticipates that this proposition will be generally acceptable to the profession, to the writers, and to the editors and publishers of magazines throughout the country.

To carry out this purpose he has engaged Augustus K. Gardner, A. M., M. D., late Professor of Diseases of Females, in the New York Medical College, well known to the profession by his numerous popular writings, who will personally edit this additional "Half-Yearly Digest of the Medical Sciences" in the United States.

